# Revealing fosfomycin primary effect on *Staphylococcus aureus *transcriptome: modulation of cell envelope biosynthesis and phosphoenolpyruvate induced starvation

**DOI:** 10.1186/1471-2180-10-159

**Published:** 2010-06-01

**Authors:** Marko Petek, Špela Baebler, Drago Kuzman, Ana Rotter, Zdravko Podlesek, Kristina Gruden, Maja Ravnikar, Uroš Urleb

**Affiliations:** 1 Department of Biotechnology and Systems Biology, National Institute of Biology, Večna pot 111, Ljubljana, SI-1000, Slovenia; 2 Novartis BPO Mengeš, Lek Pharmaceuticals d.d., Kolodvorska 27, Mengeš, SI-1234, Slovenia; 3 Department of Biology, Biotechnical Faculty, University of Ljubljana, Jamnikarjeva 101, Ljubljana, SI-1000, Slovenia

## Abstract

**Background:**

*Staphylococcus aureus *is a highly adaptable human pathogen and there is a constant search for effective antibiotics. Fosfomycin is a potent irreversible inhibitor of MurA, an enolpyruvyl transferase that uses phosphoenolpyruvate as substrate. The goal of this study was to identify the pathways and processes primarily affected by fosfomycin at the genome-wide transcriptome level to aid development of new drugs.

**Results:**

*S. aureus *ATCC 29213 cells were treated with sub-MIC concentrations of fosfomycin and harvested at 10, 20 and 40 minutes after treatment. *S. aureus *GeneChip statistical data analysis was complemented by gene set enrichment analysis. A visualization tool for mapping gene expression data into biological pathways was developed in order to identify the metabolic processes affected by fosfomycin. We have shown that the number of significantly differentially expressed genes in treated cultures increased with time and with increasing fosfomycin concentration. The target pathway - peptidoglycan biosynthesis - was upregulated following fosfomycin treatment. Modulation of transport processes, cofactor biosynthesis, energy metabolism and nucleic acid biosynthesis was also observed.

**Conclusions:**

Several pathways and genes downregulated by fosfomycin have been identified, in contrast to previously described cell wall active antibiotics, and was explained by starvation response induced by phosphoenolpyruvate accumulation. Transcriptomic profiling, in combination with meta-analysis, has been shown to be a valuable tool in determining bacterial response to a specific antibiotic.

## Background

*Staphylococcus aureus *is a common human pathogen. It is known to be highly adaptable, as shown in the rapid development of resistance to most known antibiotics. Much research in the last decade has been devoted to discovering new broad-spectrum antibiotic agents. A large proportion of effective antibiotics act on the cell wall which has been taken as an adequate target in the development of new drugs. Most cell wall active antibiotics in clinical use, for example β-lactams and glycopeptides, act by inhibiting late steps of peptidoglycan synthesis on the outer side of the cell membrane. The enzymes that catalyze the intracellular part of the peptidoglycan synthesis pathway, muramyl peptide ligases (Mur enzymes), are also good candidates for antibiotic drug targeting, because human cells do not synthesize similar enzymes. Inhibition of these enzymes causes substantial impairment of bacterial cell wall biosynthesis which, at higher doses of inhibitor, leads to decreased cell growth and to cell lysis. However, only two antibiotic agents targeting Mur enzymes are in clinical use, fosfomycin and cycloserine. Fosfomycin is a potent irreversible inhibitor of MurA, an enolpyruvyl transferase that catalyses the condensation of uridine diphosphate-N-acetylglucosamine with phosphoenolpyruvate (PEP) [1]. This reaction is the first step in the peptidoglycan biosynthesis pathway.

Genome-scale expression profiling, using microarray technology, can be used to determine potential drug targets [2]. The *Staphylococcus aureus *microarray meta-database (SAMMD, [3]) contains sets of differentially expressed genes, identified by published *S. aureus *expression profiling experiments. This database simplifies comparison of experimental data and provides a quick overview of published experiments for this bacterium.

Our goal is to develop a platform for transcriptional profiling of new Mur ligase inhibitors. As a reference, the transcription profile was determined for the well characterized inhibitor of MurA ligase, fosfomycin. We have focused on the pathways and processes primarily affected by fosfomycin. In contrast to other genome-wide profiling studies of pathogen responses to antimicrobial substances, we have studied the response to low concentrations of antimicrobial agent early after its addition. An innovative data analysis approach, complemented by newly devised visualization tools, pathway analysis and meta-analysis of similar experiments, enabled us to identify differentially expressed gene groups and pathways, and to conclude that the response of the bacterium to fosfomycin is not only time but also concentration dependent.

## Results and discussion

**Figure 1:**
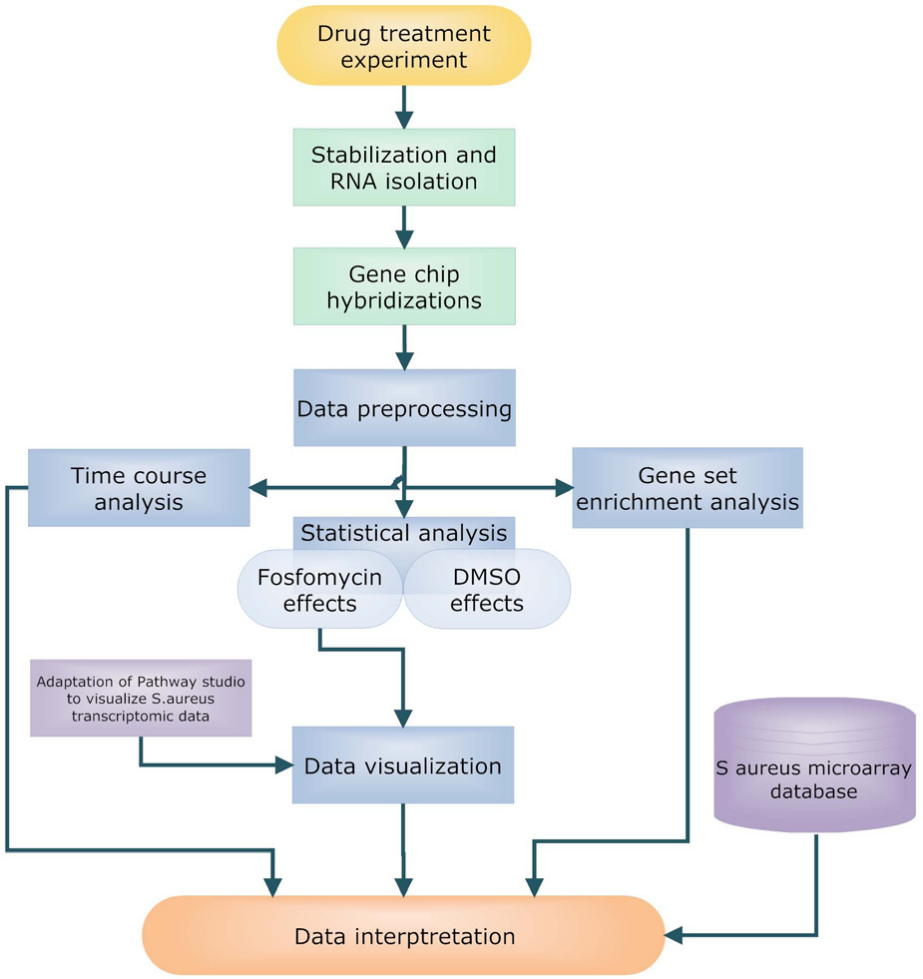
**Experimental workflow outlining the microarray data analysis procedure**.

The experiment was designed to enable detection of primary effects of fosfomycin treatment, as opposed to the cell death related effects observed after prolonged exposure to high drug concentrations. The longest time of exposure was chosen to be 40 min, which is approximately one cell cycle. Two concentrations of fosfomycin were used, 1 μg/ml and 4 μg/ml, which affected bacterial growth only slightly (results not shown). The samples were processed and the data obtained analyzed according to strict protocol as shown schematically in Figure 1.

### Time and concentration dependent effects of fosfomycin

**Figure 2:**
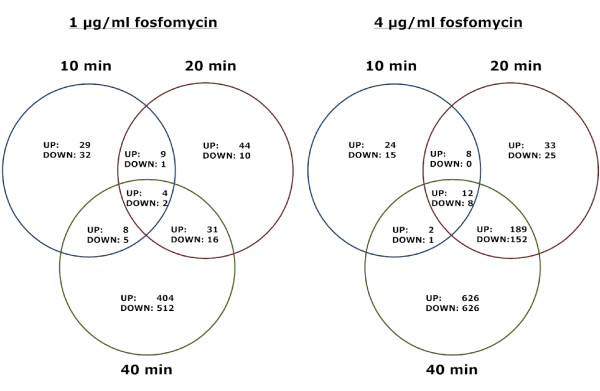
**Venn diagrams of differentially expressed genes in fosfomycin treated vs. control *S. aureus *cultures**. Circles show numbers of differentially expressed genes (UP- upregulated, DOWN- downregulated) 10, 20 and 40 minutes after treatment with 1 μg/ml (left) and 4 μg/ml (right) of fosfomycin.

**Figure 3:**
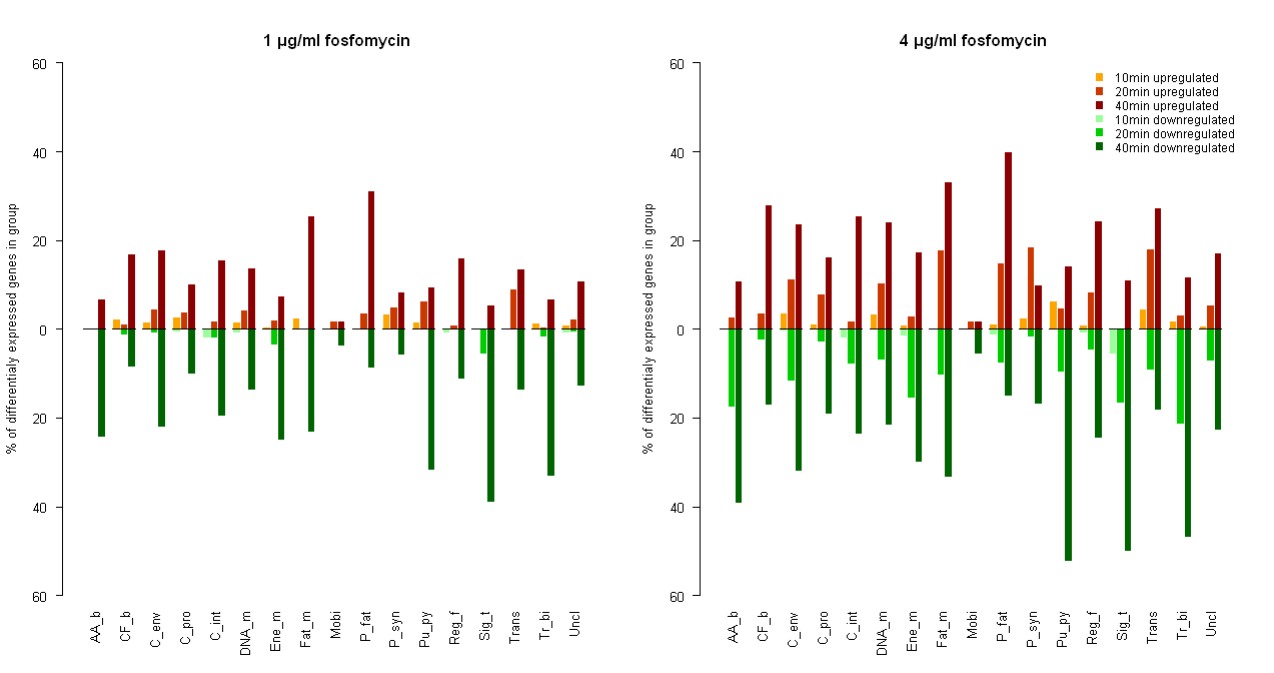
**Differentially expressed genes corresponding to TIGRFAM protein superfamilies**. The percentage of differentially expressed genes (upper panel - upregulated genes, lower panel - downregulated genes) vs. total number of genes in TIGRFAM protein superfamilies (AA_b: Amino acid biosynthesis; CF_b: Biosynthesis of cofactors, prosthetic groups, and carriers; C_env: Cell envelope; C_pro: Cellular processes; C_int: Central intermediary metabolism; DNA_m: DNA metabolism; Ene_m: Energy metabolism; Fat_m: Fatty acid and phospholipid metabolism; Mobi: Mobile and extrachromosomal element functions; P_fat: Protein fate; P_syn: Protein synthesis; Pu_py: Purines, pyrimidines, nucleosides, and nucleotides; Reg_f: Regulatory functions; Sig_t: Signal transduction; Trans: Transcription; Tr_bi: Transport and binding proteins; Uncl: Unclassified) at 10, 20 and 40 minutes after treatment with 1 μg/ml (left) and 4 μg/ml (right) of fosfomycin.

The profile of differentially expressed genes varied substantially with time following treatment with fosfomycin. After ten minutes, only a small proportion of genes were significantly differentially expressed (Figure 2). This first time point was too short to detect global changes at the level of gene expression. The reaction to fosfomycin became more evident after 20 min and 40 min of incubation. The greatest number of differentially expressed genes was found at 4 μg/ml fosfomycin concentration, after 40 min incubation (t40c4) (Figure 2 and Figure 3). Not surprisingly, at both concentrations, the later time points were more similar to one another than to the time point 10 min of incubation in terms of common differentially expressed genes (Figure 2).

The concentration dependence of the transcriptome response was also observed at the individual gene level. For example, alanine racemase gene SA1231, some transporter genes (*opp2B*, SA1183, SA1972, *msmX*, SA0207, *malF*) and amino acid biosynthesis genes *dhoM *and *hisC *were significantly differentially expressed only at higher concentrations of fosfomycin (see Additional file 1).

### Metabolic pathways affected by fosfomycin treatment

Analysis of gene groups and metabolic pathways is suitable for biological interpretation of microarray analysis results, where grouping is essential to retain the overview. We have chosen TIGRFAM functional classification to group *S. aureus *genes by the known or predicted biochemical role of the protein they encode. The greatest proportion of differentially expressed genes belong to the groups "cell envelope", "transport and binding proteins" and "energy metabolism", indicating that these were the processes affected most by fosfomycin (Figure 3). A global transcriptional response became evident after 20 min of incubation. Interestingly, mainly the same processes were affected at both concentrations.

The results of pathway analysis obtained by the different approaches - one classifying differentially expressed genes (Figure 3), the other comparing the whole expression profiles by gene set enrichment analysis (GSEA) (Table 1) - were similar, confirming the biological significance of the results. Both approaches show that fosfomycin downregulated genes for amino acid biosynthesis, transport, and energy metabolism, but upregulated those for protein synthesis and protein fate (protein modification, trafficking, repair, and folding). Interestingly, GSEA shows that for cell envelope genes, purine and pyrimidine biosynthesis, and for regulatory genes, the switch in transcription regulation, occurred 20 min after treatment. The upregulation of genes for cell division after 40 min of treatment (Table 1) is important, since many components of this process are involved in cell envelope biosynthesis.

**Table 1:** Enriched gene sets after 10, 20 and 40 minutes of treatment with fosfomycin.

	Downregulation			Upregulation		
Gene set	10 min	20 min	40 min	10 min	20 min	40 min
AMINO ACID BIOSYNTHESIS_ASPARTATE FAMILY	**0.000**	**0.003**	**0.005**			
AMINO ACID BIOSYNTHESIS_OTHER		0.171				
TRANSPORT AND BINDING PROTEINS_AMINO ACIDS, PEPTIDES AND AMINES		**0.000**	**0.010**			
TRANSPORT AND BINDING PROTEINS_CARBOHYDRATES, ORGANIC ALCOHOLS, AND ACIDS		0.090	**0.008**			
TRANSPORT AND BINDING PROTEINS_CATIONS AND IRON CARRYING COMPOUNDS		0.078	0.228			
TRANSPORT AND BINDING PROTEINS_UNKNOWN SUBSTRATE		0.092	**0.022**			
ENERGY METABOLISM_AMINO ACIDS AND AMINES		0.135	**0.008**			
ENERGY METABOLISM_ATP-PROTON MOTIVE FORCE INTERCONVERSION, BIOSYNTHESIS AND DEGRADATION OF POLYSACCHARIDES, PYRUVATE DEHYDROGENASE			**0.005**			
ENERGY METABOLISM_GLYCOLYSIS_GLUCONEOGENESIS		0.088	0.238			
ENERGY METABOLISM_SUGARS AND TCA CYCLE		0.077	0.089			
SIGNAL TRANSDUCTION_PTS		**0.033**	**0.008**			
CELL ENVELOPE_BIOSYNTHESIS AND DEGRADATION OF MUREIN SACCULUS AND PEPTIDOGLYCAN					0.068	**0.015**
CELL ENVELOPE_BIOSYNTHESIS AND DEGRADATION OF SURFACE POLYSACCHARIDES AND LIPOPOLYSACCHARIDES		**0.000**	**0.009**	0.228		
CELL ENVELOPE_OTHER		0.087				
CELLULAR PROCESSES_CELL DIVISION					0.238	0.051
CELLULAR PROCESSES_PATHOGENESIS		0.237				
CELLULAR PROCESSES_TOXIN PRODUCTION AND RESISTANCE			0.068			
CENTRAL INTERMEDIARY METABOLISM_NITROGEN METABOLISM AND AMINO SUGARS					**0.046**	
CENTRAL INTERMEDIARY METABOLISM_OTHER		0.140				
PURINES, PYRIMIDINES, NUCLEOSIDES, AND NUCLEOTIDES		**0.000**		**0.036**		
REGULATORY FUNCTIONS_OTHER						0.169
PROTEIN SYNTHESIS_TRNA AMINOACYLATION		0.083				
PROTEIN FATE_DEGRADATION OF PROTEINS, PEPTIDES, AND GLYCOPEPTIDES					0.238	0.220
PROTEIN FATE_PROTEIN AND PEPTIDE SECRETION AND TRAFFICKING					0.071	**0.020**
PROTEIN FATE_PROTEIN MODIFICATION AND REPAIR_PROTEIN FOLDING AND STABILIZATION					0.132	**0.000**
PROTEIN SYNTHESIS_RIBOSOMAL PROTEINS: SYNTHESIS AND MODIFICATION_TRANSLATION FACTORS				**0.001**		**0.005**
PROTEIN SYNTHESIS_TRNA AND RRNA BASE MODIFICATION						0.241
TRANSCRIPTION						**0.030**
DNA METABOLISM						0.249

Downregulation corresponds to negative correlation and upregulation corresponds to positive correlation with the fosfomycin concentration. Numbers show false discovery rates (FDR). Only gene sets with FDR < 0.25 in at least one time point are shown; bold is used when FDR < 0.05.

**Figure 4:**
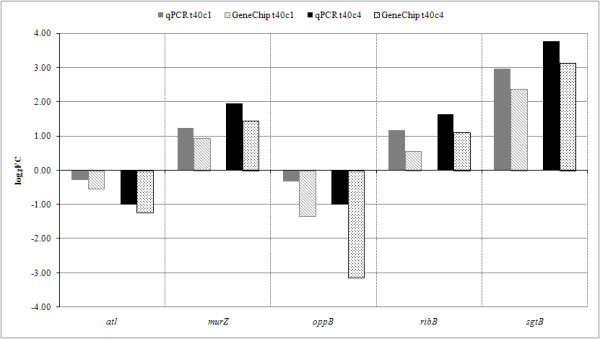
**Verification of microarray results by qPCR**. Differential expression of *atl*, *murZ*, *oppB*, *ribB*, and *sgtB *genes was measured after 40 min of treatment with 1 μg/ml (t40c1) and 4 μg/ml (t40c4) of fosfomycin. The histograms show log_2 _fold changes (log_2_FC). The filled bars show qPCR data and the patterned bars microarray data.

To strengthen the reliability of the microarray data, qPCR analysis was performed for five differentially expressed genes - two peptidoglycan biosynthesis genes, *murZ *and *sgtB*, autolysin gene *atl*, cofactor biosynthesis gene *ribB *and oligopeptide transporter gene *oppB *(Figure 4).

### Cell envelope synthesis is strongly affected by fosfomycin treatment

The GSEA results showed that specific subgroups of genes in the cell envelope group were regulated differently (Table 1). Genes involved in murein and peptidoglycan biosynthesis, including teichoic acid biosynthesis genes, were upregulated, while surface polysaccharide metabolism genes were downregulated.

To interpret the changes in gene expression we visualized the data in Pathway Studio software. Since the peptidoglycan biosynthesis pathway is not complete in the existing metabolic network [4], the pathway was complemented with literature data. The .gpc file (Additional file 2) can be used by the scientific comunity to interpret gene expression data, enabling ready visual comparison of experimental results from different studies.

**Figure 5:**
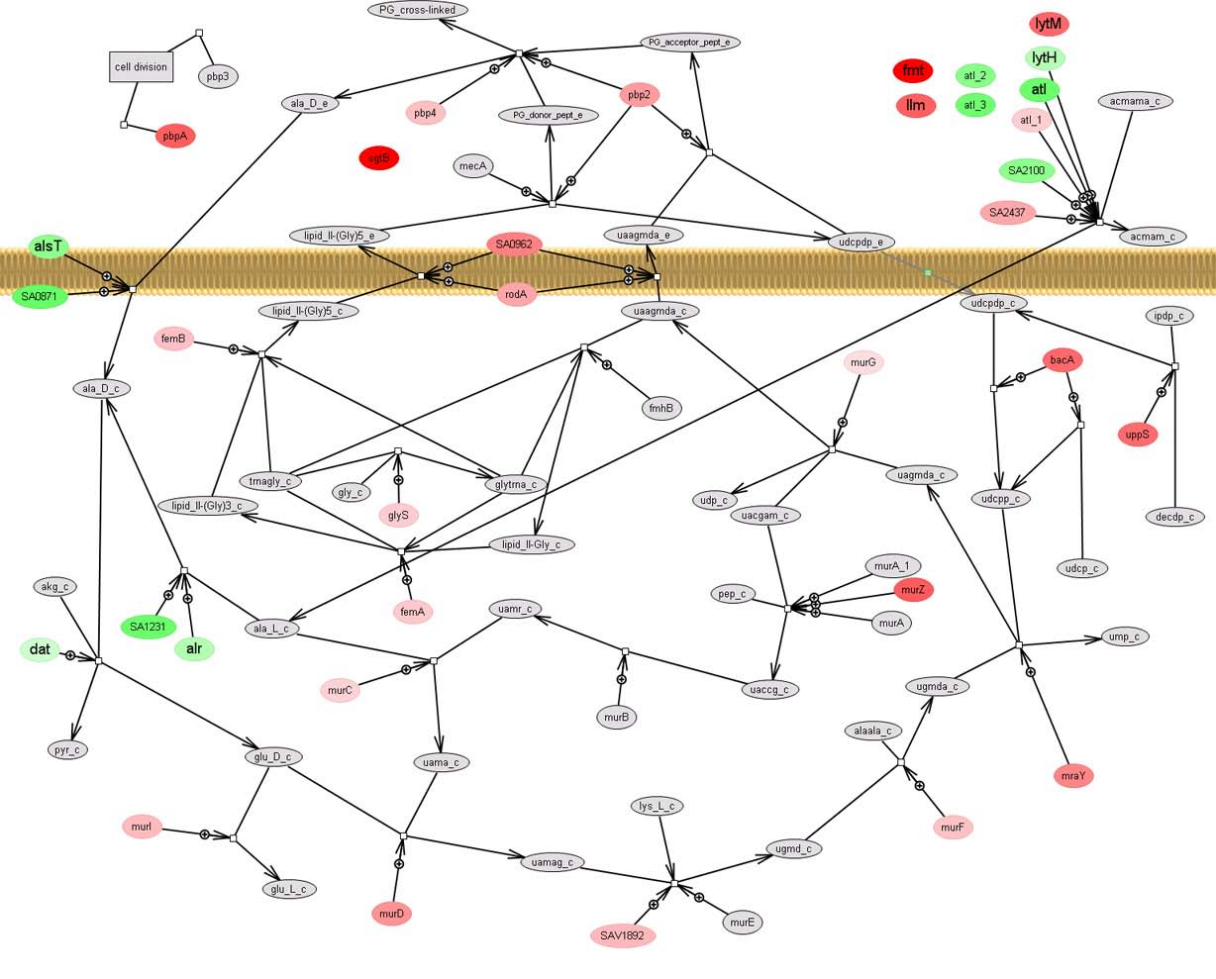
**Visualization of *S. aureus *peptidoglycan metabolic pathway**. Node colours correspond to fold changes of differentially expressed genes 40 min after treatment with 4 μg/ml of fosfomycin (red - upregulated, green - downregulated, grey - genes not differentially expressed). Metabolites are represented by grey-shaded nodes without the plus sign on the connecting arcs.

Fosfomycin caused weak upregulation of several *mur *genes (*murIDZ, mraY*) that encode enzymes involved in the first step of peptidoglycan biosynthesis (Figure 5). This was observed at time point t40c4 only. The most strongly induced of the *mur *genes was that encoding MurZ, a MurA homologue enzyme. Fosfomycin inhibits both MurA and MurZ, which are essential to Gram positive bacteria [5]. Nevertheless, the *murA *gene (with two probe sets on the chip: MurA, MurA_1; Figure 5) was not found to be significantly differentially expressed. Interestingly, some genes encoding enzymes acting in the final phases of peptidoglycan synthesis - *pbpA*, *bacA*, and *sgtB *- were more induced than the gene encoding the target enzyme (Figure 5). This suggests that inhibition of MurA and MurZ affects transcription of the whole metabolic pathway. In contrast to *Escherichia coli*, peptidoglycan biosynthetic genes in *S. aureus *are distributed evenly throughout the chromosome and are regulated independently. As shown by Sobral et al. [6], there is a striking complexity of transcription level links that connect a large number of diverse cellular functions to any particular step in cell wall synthesis.

Autolysin coding genes *atl*, *lytH*, SA0423, and SA2100 were downregulated at t40c4, whereas *lytM *was upregulated by fosfomycin at that point (Figure 5) suggesting the prevention of further degradation of peptidoglycan. As well as in cell wall stress, gene *atl *has been found to be downregulated in acid shock [7], SOS response and, cold shock, but upregulated in stringent response [8].

A set of *S. aureus *genes responding to cell wall active antibiotics, termed the "cell wall stress stimulon", were first described by Utaida et al. [9]. They showed an orchestrated response following treatment with antibiotics acting at different stages of cell wall biosynthesis, either intra- (D-cycloserine) or extra-cellularly (vancomycin, oxacillin, bacitracin), at different exposure times and concentrations. The qualitative comparison of differential expression of the cell wall stress stimulon genes in our and previously described studies is presented in Table 2. Despite the different experimental conditions, the cell wall stress stimulon genes react in a similar manner, independently of the bacterial strain and incubation time. Moreover, in our experiment, the transcriptional response of these genes is seen to be time and concentration dependent (Table 2). Their expression is controlled mainly by the *vraSR *two component system and it has been shown that the VraSR regulon is induced specifically only by cell-wall-active antibiotics [10]. Fosfomycin strongly induced the *vraS *(Table 2) and vraR (Additional file 1) genes and many of the genes they regulate - not only cell wall synthesis genes but also those for chaperones, heat shock proteins and osmoprotectant transporters. The *rib *and *ure *operons, involved in cofactor biosynthesis and urea degradation and, which were induced by some cell-wall-active antibiotics, were also induced at the latest time point in our experiment.

**Table 2:** Expression of "cell wall stress stimulon" genes: comparison of current study with published results in the SAMMD.

N315 LOCUS^*a*^	Gene Name^*b*^	Expression fold change^*c*^							Gene Product Description^*e*^	TIGR Functional Group
				
		t10c1	t20c1	t40c1	t10c4	t20c4	t40c4	Cell wall active antibiotics^*d*^		
SA0909	fmt			2.65		1.83	3.23	+	Fmt, autolysis and methicillin resistant-related protein	Cell envelope
SA1549				1.38		0.63	1.87	+	hypothetical protein, similar to serine proteinase	Protein fate
SA1659	prsA			1.57		0.94	1.89	+	peptidyl-prolyl cis/trans isomerase homolog	Protein fate
SA1691	sgtB		0.37	2.37		1.31	3.14	+	hypothetical protein, similar to penicillin-binding protein 1A/1B	Cell envelope
SA1701	vraS		0.28	2.05		1.21	2.93	+	two-component sensor histidine kinase	Cellular processes
SA1702				2.25		1.29	3.34	+	conserved hypothetical protein	Unclassified
SA1703				2.63		1.47	3.54	+	hypothetical protein	Unclassified
SA1712				0.69		0.41	1.60	+	conserved hypothetical protein	Unclassified
SA1926	murZ			0.94		0.51	1.45	+	UDP-N-acetylglucosamine 1-carboxylvinyl transferase 2	Cell envelope
SA2103				1.58		0.87	2.11	+	hypothetical protein, similar to lyt divergon expression	Regulatory functions
SA2146	tcaA		0.27	2.07		1.27	2.69	+	TcaA protein	Energy metabolism
SA2220				0.95		0.47	1.48	+	hypothetical protein	Energy metabolism
SA2221				1.92		0.96	2.59	+	hypothetical protein	Unclassified
SA2297							0.37	+	hypothetical protein, similar to GTP-pyrophosphokinase	Unclassified
SA2343		-0.73	2.11	7.08		5.50	7.62	+	hypothetical protein	Unclassified

SA0423				-0.47			-1.34	-	hypothetical protein, similar to autolysin (N-acetylmuramoyl-L-alanine amidase)	Unclassified
SA0905	atl			-0.54			-1.24	-	autolysin	Cell envelope
				-0.51			-1.19			

*^a ^S. aureus *N315 genome ORF locus.

*^b ^*Previously described gene name.

*^c ^*Gene expression log_2 _fold change of treated vs. non-treated bacteria. Abbreviations correspond to experimental design points.

*^d ^*Previously reported expression increase (+) or decrease (-) of cell-wall-antibiotic treated vs. non-treated bacteria.

*^e ^*Gene product functional annotation.

### A transcriptional response specific to MurA inhibition

**Figure 6:**
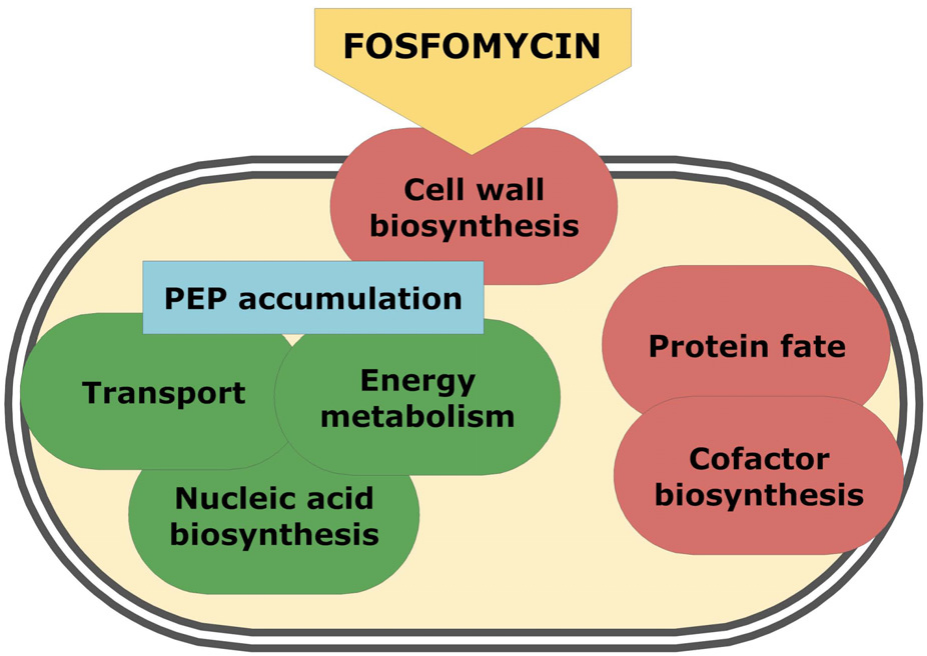
**Fosfomycin effects on *S. aureus *metabolism supported by transcriptional data in this study**. Processes in red ovals were induced and those in green ovals were repressed by fosfomycin treatment.

We performed a meta-analysis to identify groups of genes responding differently to fosfomycin and to other antibiotic treatments. For example, the transcriptional response to ciprofloxacin [11], an inhibitor of bacterial DNA gyrase, is clearly different from that of fosfomycin, because the cell wall stress stimulon genes were not activated. Similarly, the transcriptional profile of the antiseptic compound triclosan, that targets fatty acid biosynthesis [12], confirms the specificity of the cell wall stress response. The effects of fosfomycin on *S. aureus *metabolism, supported by our transcription data, are schematized in Figure 6. The inhibition of MurA causes accumulation of its substrate phosphoenolpyruvate (PEP) which is known to act as a carbon starvation signal. PEP accumulation was shown to be responsible for downregulation of several central metabolism genes and nucleic acid biosynthesis genes in different organisms including bacteria [13]. A downregulation of *pur *and *pyr *operons was observed at the latest time point. Downregulation of both operons has also been reported in the SOS response [8], acid-shock response [7], ciprofloxacin response [11] and in the *S. aureus *MurF underexpression mutant [6].

In order to reach target enzymes MurA and MurZ, fosfomycin has to cross the cell membrane. Because of its hydrophilic nature it uses the active transport systems (ABC transport proteins), specifically the L-α-glycerophosphate and the glucose-6-phosphate uptake systems [1]. The PEP phosphotransferase system (PTS) mediates the uptake and phosphorylation of carbohydrates and controls metabolism in response to carbohydrate availability, and can therefore affect the whole cell metabolic rate [14]. GSEA shows that PTS was downregulated by fosfomycin 20 and 40 minutes after treatment. This downregulation could be a defense mechanism against the influx of fosfomycin. It has been reported that PTS mutant bacteria are highly resistant to fosfomycin [15] and that some fosfomycin-resistant *E. coli *isolates have altered *glpT *and/or *uhp *transport systems [16]. The downregulation of PTS genes can also contribute to PEP accumulation [13]. As shown in Figure 3 and Table 1, transport processes in general were significantly downregulated. The majority of differentially expressed genes in this group encode proteins that transport oligopeptides (*opp *genes), amino acids, sugars, polyamines (*potABCD*) and cations into the cell. Genes encoding iron transport and binding proteins, belonging to the Isd system, were also downregulated similarly as in a MurF underexpression mutant study [6]. However, a small proportion of transport genes were upregulated, including some amino acid and oligopeptide carrier genes and the sodium/hydrogen exchanger genes *mnhBCDEG*.

The energy metabolism group, consisting of genes involved in sugar metabolism, amino acid degradation and TCA cycle, were generally downregulated (Figure 3), consistent with a starvation response. The downregulated amino acid metabolism genes include *met *and *dap *operons; additionally, the aspartate family was shown to be significantly downregulated by GSEA (Table 1). Upregulated amino acid metabolism genes include genes involved in cysteine biosynthesis and synthesis of cystathionine. Various tRNA synthetases, probably connected to amino acid biosynthesis, were also downregulated.

Strong downregulation of virulence genes by fosfomycin was observed, especially 40 min after treatment. These genes include *hla, spa, aur, sspABC *and 16 *cap *genes (*capA - capF*) encoding capsular polysaccharide synthesis enzymes. Capsular genes were also downregulated in the SOS response [8], but upregulated by cycloserine treatment [9], *sigB *mutant [17] and biofilm forming *S. aureus *[18]. It has been shown that *cap *genes and various virulence factors are regulated by Sae and Agr global regulatory proteins. It was shown that Agr causes induction, and Sae repression, of *cap *genes [19,20], but in our experiments none of these regulatory genes were differentially expressed.

## Conclusions

A pathway-based approach enabled us to determine that the response of *S. aureus *to fosfomycin is not only time but also concentration dependent, and that the major transcriptional switch occurred after 20 to 40 min of treatment. The fosfomycin response was similar to those of other cell-wall-active antibiotics in the cell envelope pathway and the cell wall stress stimulon genes. However, in contrast to previously described cell-wall-active antibiotic treatments, we have identified several pathways and genes downregulated by fosfomycin, such as transport, nucleic acid biosynthesis, energy metabolism and virulence genes. The downregulation of these pathways was explained by a starvation response induced by PEP accumulation. We have shown that transcriptomic profiling, in combination with meta-analysis, is a valuable tool in determining bacterial response to a specific antibiotic.

## Methods

### Bacterial growth conditions

*Staphylococcus aureus*, strain ATCC 29213 was cultured in a small volume of cation-adjusted Mueller-Hinton broth medium (Sigma-Aldrich) and grown in Erlenmeyer flask on a gyratory shaker (200 rpm) at 37°C. The overnight culture was diluted 100-fold in 300 ml of medium and grown under the same conditions in 1-L Erlenmeyer flasks until OD_600 _reached 0.3, which corresponded to the early exponential stage of growth.

### Antibiotic treatment

With the potential of testing new chemical entities in mind, the experiment was designed to allow substances slightly soluble in water to be tested. Fosfomycin (Sigma) was diluted in DMSO (Sigma) to give final concentrations of 5% DMSO with 1 (c1) and 4 (c4) μg/ml of fosfomycin. DMSO alone was added to the control cultures (c0), to normalize the effects of DMSO treatment.

Appropriate fosfomycin concentrations were determined in a preliminary growth study (data not shown). Growth rate (measured as OD) and proportion of live cells determined with the LIVE/DEAD BacLight™ Bacterial Viability Kit (Invitrogen) were monitored for a range of concentrations from 1 to 1024 μg/ml. For the microarray experiments concentrations were selected that did not affect bacterial growth in the first few hours after treatment. The experiment was repeated four times, from four independently grown bacterial inoculates, thus yielding 40 samples.

### Sampling and RNA preparation

The bacterial culture (prepared as described above) was divided into 10 flasks (19 ml per flask) containing previously prepared fosfomycin solutions. Cultures were grown as described above and sampled (7 ml per flask) at the time of treatment (t0) and 10 (t10), 20 (t20) and 40 minutes (t40) after treatment. The OD of each culture was measured immediately before sampling (data not shown) and the cultures were stabilized using RNAprotect Bacteria Reagent (Qiagen), following the manufacturers protocol. The bacterial pellets were stored at -80°C.

RNA was isolated from bacterial pellets by enzymatic cell wall lysis [21] followed by RNeasy Mini Kit (Qiagen) purification. Two hundred μl of lysis buffer (20 mM TRIS HCl, 50 mM EDTA, 200 g/l sucrose, pH 7.0), containing lysostaphin (Sigma; 15 μg/μL) was added to the cell pellet and incubated on ice for 20 minutes. The lysate was transferred to a water bath at 37°C for 3 minutes. After incubation, 200 μl of 2% SDS and 7 μl of proteinase K were added and the lysate incubated at room temperature for 15 minutes. 800 μl of the RLT buffer (from RNeasy Kit) was added to the lysate, vortexed vigorously and sonicated for 5 minutes at 56°C. After the addition of 600 μl of absolute ethanol, the lysate was transferred to the RNeasy Mini columns and centrifuged until all the lysate was used. The remaining steps were as described in RNeasy Mini Kit manufacturer's protocol. The elution was performed twice with pre-heated (60°C) water and 5 minutes incubation time. To remove remaining genomic DNA, total RNA samples were treated with DNase I (Deoxyribonuclease I, amplification grade, Invitrogen), as recommended by manufacturer, only with lower optimized DNase concentration of 0.25 U per μg of total RNA. The RNA was purified and concentrated using RNeasy Min Elute Kit (Qiagen). Finally the RNA was checked for quality and quantity using absorbance measurements (Nanodrop) and agarose gel electrophoresis (data not shown). Two samples did not meet the quality demands and were not used for microarray hybridization.

### Microarray hybridization

RNA was labelled and hybridized to GeneChip^® ^*S. aureus *Genome Arrays (Affymetrix) according to the GeneChip^® ^Expression Analysis Technical Manual, the section for prokaryotic antisense arrays. Targets were prepared by cDNA synthesis with random primers, RNA degradation, cDNA purification and fragmentation, followed by terminal labelling with biotin. Labelled cDNA was hybridized on the microarrays, which were subsequently washed, stained and scanned.

### Quality control and statistical data analysis

Data was analysed with bioconductor (R version 2.10.0; http://www.bioconductor.org) packages affy [22], gcrma [23] and limma [24]. Quality control of the microarray consisted of visual inspection of various diagnostic plots, namely boxplots of transcript intensities, image plots of arrays and MA plots of raw data. Additionally, parameters from the Affymetrix software were evaluated. Moreover, RLE (Relative Log Expression) and NUSE (Normalized Unscaled Standard Error) plots were constructed [25]. Of 38 analyzed arrays, one did not meet the quality requirements and was therefore excluded from further analysis.

Data pre-processing and expression value calculation were carried out using two procedures, yielding 2 separate datasets. In the first, a combination of rma convolution method for background adjustment [26], invariantset for normalization [27], pm correction as from the mas manual, and liwong method summarization [27,28] were applied. In the second procedure, all the pre-processing steps were performed simultaneously using gcrma [23].

In order to find differentially expressed genes a statistical model was formulated (p < 0.05) to compare gene expression in bacteria exposed to fosfomycin concentrations c1 and c4 with that of the control (c0) at a given time point. To decrease false discovery rate, the results coming from different pre-processing procedures were combined and only the intersection of genes, differentially expressed following both procedures were taken into account for the biological interpretation of the results [29].

### Pathway analysis

Biochemical reactions from *S. aureus *metabolic network reconstruction *i*SB619 [4] were obtained from BIGG database http://bigg.ucsd.edu/ and coupled with TIGR *S. aureus *annotation [30] downloaded from TIGR CMR database http://cmr.tigr.org/tigr-scripts/CMR/CmrHomePage.cgi. Pathway database and expression profiles for all experimental time points were imported to Pathway Studio software (version 4.0; Ariadne Genomics Inc). Differentially expressed genes were queried for presence in metabolic network. Pathways constructed in Pathway Studio were examined and interpreted manually. Pathway Studio .gpc file is available as Additional file 2.

Gene set enrichment analysis (GSEA) [31] was applied to search for groups of genes involved in the same processes (gene sets) that were altered significantly by fosfomycin treatment. Individual GSEA was performed for a data set including control and both fosfomycin treatment concentrations (1 and 4 μg/ml) for the selected time point. Gcrma-normalized data was filtered for signal intensity greater than 10. The signal intensities from the same time point were overlapped on 40 gene sets (see Additional file 3) based on TIGR *S. aureus *annotation [30] and measured for the enrichment of genes at the top or bottom of the gene list to determine their correlation with the logarithm of fosfomycin concentration (gene set's phenotype). The GSEA parameters used included: Pearson metric and gene set size restrictions, 10 minimum, 500 maximum. Gene sets significantly modified by fosfomycin treatment were identified using a multiple hypothesis testing FDR < 0.25. GSEA was performed for each time point (10, 20 and 40 min) at which gene expression was correlated with fosfomycin concentration. Positive correlation was interpreted as up-regulation of a gene set resulting from drug treatment; a negative correlation was interpreted as down-regulation.

### Meta-analysis: integration of gene expression data from other sources

Our experimental data was compared to other publicly available *S. aureus *transcriptomic data. To ease the comparison, the recently published "*Staphylococcus aureus *microarray meta-database" (SAMMD) was used [3]. The qualitative transcriptional profiles (up or downregulation) were coupled with the quantitative transcriptional profile of fosfomycin to a single spreadsheet (Additional file 1) in order to analyze the similarities and differences between different responses.

### Quantitative real-time PCR (qPCR)

The purified RNA samples from experimental points t40c0, t40c1 and t40c4 were reverse transcribed using High Capacity cDNA Reverse Transcription Kit (Applied Biosystems). The acquired cDNA was used to validate the microarray differential expression for genes listed in Table 3. All qPCR reactions were performed on a LightCycler LC480 Detection System (Roche) in 384-well plate format using universal cycling conditions (2 min at 50°C, 10 min at 95°C, followed by 50 cycles of 15 s at 95°C and 1 min at 60°C). Real-time PCR was performed in a final reaction volume of 5 μL containing 2 μL of diluted cDNA sample, 1× primer-probe mix (TaqMan^® ^Gene Expression Assay, Applied Biosystems) and 1× TaqMan^® ^Universal PCR Master Mix (Applied Biosystems). Each sample cDNA was tested for five target genes: *atl, murZ, oppB, ribB, sgtB *and the endogenous control 16S rRNA [32]. The TaqMan^® ^chemistry based primers and probes were designed and synthesized by Applied Biosystems (Table 3). Each reaction was performed in two replicate wells in two dilutions on the same 384-well plate. An automated liquid handling system (Multiprobe^® ^II plus ex, PerkinElmer) was used to prepare cDNA dilutions, to pipette cDNA samples and master mixes onto the 384-well plates.

**Table 3:** Primer and probe sequences used for qPCR analysis.

Gene Name	Forward Primer	Reverse Primer	Probe
*murZ*	TGGTCCTTCATTTGTAACTGATACGATTT	CCCATGCGCTTTAATTCTTCAACAT	CCGGAGCGTTTTAAAC
*atl*	CCCTACTACACCATCAAAACCAACA	TGTGCGACACCATTGTTTGC	ACACCGTCGAAACCAT
*ribB*	CGTGCCATGAGTGGTAACG	GTTCATCTACATGACCGAGGACAAA	ATGTCCACCAAACCTAC
*sgtB*	GAGCTTTATTTTCAACGATTAGCGACA	AATTTTTGACAACTTGTTGTGTAATGGTACTAC	CACCTTGCACATCTC
*oppB*	TTTAGGTGTTGCAGCAGCTACT	GTACAGCAAGTACAAAAGATGGTACAGA	CAACCCAAGAATTTTG

Data were analyzed using the LightCycler^® ^480 1.5.0.39 Software. The 2nd derivate method was used for all amplicons to determine Cp values. The standard curve method was used for relative gene expression quantification, and the transcript accumulation of each gene was normalized to 16S rRNA. The amplification efficiency and linear range of amplification were followed for each amplicon on each plate by analyzing a reference sample pool in four dilution steps of cDNA with two replicate wells per dilution step. Each sample was analyzed in two dilutions and two replicates per dilution step. Only samples where the ΔCp between two dilutions of target gene did not deviate by more than 0.5 from ΔCp of the reference gene were used for relative quantification. The fold changes for each experimental point were calculated as a quotient of average transcript abundances between treated and control samples from three independent biological replicates in each time point.

### Microarray dataset accession number

Microarray data analyzed in this study have been deposited in the Gene Expression Omnibus database with accession number GSE15394.

## Authors' contributions

ŠB performed the microarray experiment, participated in study design, data interpretation and helped to draft the manuscript. MP participated in data analysis (GSEA, Pathway Studio visualization), performed the qPCR analysis, interpreted the data, and drafted the manuscript. DK participated in the design of the study, sample preparation and data analysis (GSEA, pathway visualization). AR performed the statistical analysis. ZP participated in study design, data interpretation and drafting the manuscript. KG participated in the design of the study and data interpretation, coordinated the work and helped to draft the manuscript. MR and UU participated in the design of the study and helped to draft the manuscript. All authors read and approved the final manuscript.

## Supplementary Material

Additional file 1 **Summary table for differentially expressed genes**. Excel spreadsheet file summarizing the transcriptional data from our study and publicly available transcriptional profiling results from SAMMD. Click here for file

Additional file 2 **Pathway Studio metabolic network**. File containing the representation of *S. aureus *metabolic network (gpc format). The file can be viewed by Pathway Studio software http://www.ariadnegenomics.com/products/pathway-studio/. Click here for file

Additional file 3 **Gene sets used for GSEA**. Excel spreadsheet file containing gene sets generated from TIGRFAM ontology that were used to run GSEA. Click here for file
